# Community reinforcement and family training (CRAFT) - design of a cluster randomized controlled trial comparing individual, group and self-help interventions

**DOI:** 10.1186/s12889-019-6632-5

**Published:** 2019-03-14

**Authors:** Rikke Hellum, Anette Søgaard Nielsen, Gallus Bischof, Kjeld Andersen, Morten Hesse, Claus Thorn Ekstrøm, Randi Bilberg

**Affiliations:** 1 Unit of Clinical Alcohol Research, Clinical Institute, University of Southern Denmark, J.B. Winsløws vej 18, 5000 Odense, Denmark; 2 Psychiatric Department, Odense University Hospital, J.B. Winsløws vej 18, 5000 Odense, Denmark; 3 Odense Patient Data Explorative Network (OPEN), Odense University Hospital, Odense, Denmark; 4 Section of Biostatistics, University of Copenhagen, Øster Farimagsgade 5, 1014 København K, Denmark; 5 Centre for Alcohol and Drug Research, Department of Psychological and Behavioral Sciences, Aarhus University, Artillerivej 90, 2, 2300 København S, Aarhus, Denmark; 6 Department of Psychiatry and Psychotherapy, University of Lübeck, Ratzeburger Allee 160, 23562 Lübeck, Germany

**Keywords:** Community reinforcement and family training, CRAFT, Concerned significant others, Alcohol treatment, Treatment engagement, Alcohol use disorder

## Abstract

**Background:**

Around 585,000 people in Denmark engage in harmful use of alcohol with 140,000 suffering from outright alcohol dependence. The concerned significant others (CSOs) are affected by the drinking, often suffering almost as much as the person with alcohol use disorder. Community Reinforcement and Family Training (CRAFT) is aimed at CSOs who struggle unsuccessfully, in an effort to motivate their loved ones to stop drinking and seek treatment. The aims of this study are 1) To implement CRAFT interventions into the daily routine of operating Danish alcohol treatment centers 2) To investigate whether 6-week-individual CRAFT, 6-week-open group-based CRAFT or CRAFT based on self-help material, is efficient in getting problem drinkers to seek treatment for their alcohol problems 3) To investigate which of the three interventions (individual, group or self-directed CRAFT) is the most effective and in which group of population.

**Methods:**

The study is a three-arm, cluster randomized controlled trial: A: individual CRAFT, group CRAFT, and CRAFT as a self-help intervention. A total of 405 concerned significant others to persons with alcohol abuse will be recruited from 24 alcohol outpatient clinics. The participants will fill out a questionnaire regarding i.e. life quality, if the drinking person entered treatment (main outcome) and satisfaction with the intervention, at baseline and after 3 and 6 months.

**Discussion:**

We expect to establish evidence as to whether CRAFT is efficient in a Danish treatment setting and whether CRAFT is most effective at individual, group or self-help material only.

**Trial registration:**

Clinical trials.gov ID: NCT03281057. Registration date: September 13th, 2017.

## Background

It is estimated that 585,000 people in Denmark engage in harmful use of alcohol with 140,000 suffering from alcohol dependence [1]. However, only 15,000 [2] seek specialist treatment for their alcohol problem, often after they have been suffering from an alcohol use disorder (AUD) for more than 10 years [3]. In spite of the severe consequences of AUD, people with AUD (PWAUD) may not seek treatment for AUDs due to fear of subsequent stigma or being incapable of completing treatment [4], although the most commonly reported reason to not seek treatment is the belief that one should be able to deal with the drinking problem without outside help [5]. The concerned significant others (CSOs), such as spouses, parents, or adult children, are affected by the drinking, often suffering almost as much as the PWAUD him - or herself [6].

The populations of CSOs are silent populations. CSOs easily get caught up in a pattern of behaviour that is nothing short of destructive [7]. Studies have shown that CSOs suffer from symptoms such as anxiety, depression, stress, concentration difficulties, physical pain and anger because of a poor relationship with the PWAUD [8]. In addition, violence and sexual abuse are often linked to an alcohol problem [7, 9]. Indeed, compared to the general Danish population, the frequencies of such symptoms are two to three times more common both among persons with an AUD and among their partners [10, 11].

The CSO’s high degree of involvement in and focus on the state of a person with AUD in the family, may lead to the neglect of children in the family. It is estimated that 122,000 children in Denmark live in a family with drinking problems [12].

In the Danish National Health survey 2010 [13], heavy drinkers in the Region of South Denmark were asked where, if at all, they would seek help to reduce drinking. Most drinkers had no such desire, but were they to consider seeking help, the most common choice would be looking to family and friends, followed by the general practitioner. Choosing to seek specialist treatment ranked very low. For that reason, it makes excellent sense to empower the families and friends of the person suffering from an AUD, enabling them to act in ways that help PWAUDs towards seeking and receiving treatment.

### What kind of intervention is relevant for CSOs?

Five types of interventions, aimed at the CSOs, have been described: self-help groups (Al-Anon/Nar-Anon) which are part of the Minnesota treatment family [14], the Johnson Institute Intervention (a very confrontational approach) [15], general unspecific support (typically aimed at supporting the CSO only, but not addressing how to increase the likelihood of getting PWAUD into treatment) [16], The 5-step method [17] and Community Reinforcement and Family Training (CRAFT). So far, CRAFT is the only one of these methods with any evidence to support that it increases the likelihood of the PWAUD seeking treatment [18]. CRAFT was proposed in 1986 by Sisson and Azrin [19] aimed at training CSOs to become involved in the problem drinkers’ choice of treatment, helping CSOs handle situations between themselves and the problem drinkers, and finally helping the CSOs to develop specific strategies to take proper care of themselves in risk situations [20, 21].

### Craft

CRAFT is aimed at CSOs who struggle, unsuccessfully, to motivate their loved ones to stop drinking and seek treatment. CRAFT promotes active, positive participation from the CSOs in seeking to attract problem drinkers into treatment; changing CSOs’ efforts to help by developing their roles as active collaborators, supportive of the problem drinker. The underlying assumption of CRAFT is, that CSOs already have a detailed knowledge of the problem drinkers’ behaviour and that they are in a strong position to influence PWAUDs’ behaviour because of their concern and personal motivation, including getting PWAUDs to seek treatment [8]. So far, eight randomized or controlled clinical trials on CRAFT have been carried out on CSOs to alcohol dependents and drug users [16, 18–20, 22–25]. Three studies focus on alcohol problems [16, 19, 25], three studies on drug users [22–24], and two studies on both drug users and alcohol dependents [18, 20]. One of the studies on drug users studied CRAFT as a supplement to opioid-dependent adults already in treatment [24]. All studies have been carried out in the USA except one study on alcohol, which was carried out in Germany [25]. The German study compared CRAFT Immediate Intervention with waiting list and found a significantly higher engagement rate (B 1.34 SE 0.6) for the people receiving immediate intervention [25]. The American studies showed a two to three times higher impact in getting PWAUD to attend treatment after four to six CRAFT sessions with CSOs, compared with Al-Anon and Johnson Institute interventions [26]. In general, CRAFT interventions to CSOs lead to more than 60% of the PWAUDs in question to attending treatment in US [16]. Several of these studies have rather small samples from 12 to 40 CSOs [19, 20, 22] The original full intervention of CRAFT consists of 12–14 sessions [19]. Kirby et al. (2017) tested CRAFT in a four-six session intervention, where the only focus in the sessions was on Treatment Entry Training (TEnt) vs. the original CRAFT in 12–14 sessions and Al-Anon/Nar Anon (ANF), and 115 CSOs participated in the study. There was no counseling about e.g. relationship or substance use [18]. The treatment entry rate, after the intervention, was 62% for the CSOs randomized to the full CRAFT and 63% for the ones randomized to Tent, which was significantly higher than the ones receiving Al-Anon/Nar Anon (treatment entry rate 37%). This was to be expected, as the goal of Al-Anon/Nar-Anon is not to encourage treatment entry. No significant differences in mood and functioning were found between the three interventions, even though it was not a subject in the TEnt intervention.

CRAFT has, until now, been examined and shown effective in the USA and Germany. To our knowledge, studies on CRAFT have also been initiated in the Netherlands (ClinicalTrials.gov ID: NCT02510508) and Sweden (ISRCTN 38220020), but the results are not yet published. In the Netherlands researchers are performing a three-armed RCT with group, self-directed CRAFT or non-intervention addressed to CSOs to alcohol dependents. In Sweden, the effect of a five-week internet-based CRAFT program is tested versus waiting list (ISRCTN 38220020), in addition to another study on an online self-help program combined with a parent-training program for partners suffering from alcohol use disorder, versus a brief psycho-education program [27]. Moreover, a RCT study on CRAFT for CSOs with problem gamblers (CRAFT vs. treatment as usual) is currently being conducted in Sweden [28]. Earlier studies performed are based on small populations, and no study of CRAFT has been performed in Denmark so far.

Furthermore, to our knowledge, only one effectiveness study, performed in operating treatment institutions and as part of routine praxis, has been conducted [29]. The study from Dutcher et al., 2009, tested CRAFT in a community treatment center in the USA. Altogether 99 CSOs were concerned about alcohol abusers, whereas, for all CSOs, 55% of the treatment-refusing abusers entered treatment after 6 months. Among the CSOs, who completed at least four sessions on CRAFT, or the ones who engaged the abuser to treatment, 65% entered treatment. Further, effectiveness studies outside the USA are still essential before large-scale implementation.

The efficacy studies and the effectiveness studies performed, so far, indicate that CRAFT is effective for CSOs towards getting the drinking person into treatment and to improve the quality of life of the CSO and the relationship between the drinking person and the CSO [16, 18–20, 22–25]. Whether CRAFT delivered in group, individual or as self-help material is equally effective is, however, still unknown. The study of Manuel et al. indicated, that CRAFT in group condition may be just as effective as individual CRAFT, but the study was indeed small and did not compare the two settings directly. In the study of Manuel et al., 40 CSOs were randomized to either group CRAFT or self-directed CRAFT, and 60% of the CSOs in group CRAFT had their loved ones enter treatment, and for the self-directed CRAFT the result was 40% after six months [20]. Hence, the findings were promising, but not conclusive or significant.

Furthermore, groups can be organized as closed groups or open groups. In closed groups, all CSOs start at the same time and no new members are enrolled, once the treatment has begun. An open group can start when a minimum of two members are enrolled in the study and new members are included continually until the maximum of group members has been reached. When testing CRAFT in a closed group format, Manuel et al. experienced challenges with the start-up, because it took up to one month to gather enough CSOs to start a group [20]. Compared to closed groups, open groups can be joined without a waiting period. Furthermore, an open group format may create opportunities for senior members in the group to share experiences and advice with newcomers [20]. However, an open group format may also be negatively affected by a constant influx and outflux of people in the group, in addition to not all group members receiving the sessions in a logical order. A general strength of group therapy, in proportion to individual and self-help, may be that the CSOs meet like-minded individuals and are able to share similar experiences and feelings and support each other.

### Aim and hypotheses

The aim of this study is to implement CRAFT interventions into the daily routine of Danish community alcohol treatment centers, and investigate whether 6 week-individual CRAFT, 6 week-open group-based CRAFT or based on CRAFT self-help material only, is efficient in getting problem drinkers to seek treatment for their alcohol problems – and which of the three interventions (individual, group or self-directed CRAFT) is the most effective.

#### Hypotheses

1. CSOs, randomly assigned to either individual CRAFT or to open group CRAFT, will significantly more often be able to motivate their drinking relative to enter treatment compared to CSOs, randomly assigned to the control condition (self-directed CRAFT).

2. We hypothesize that six sessions of group CRAFT improve the quality of life and psychological functioning of CSOs significantly more than individual CRAFT and self-directed CRAFT.

## Methods

### Design

The study is a cluster randomized controlled trial, carried out in public alcohol treatment facilities. All public alcohol treatment centers in Denmark have been invited to participate in a partnership with the Unit of Clinical Alcohol Research at the University of Southern Denmark. In all, 61 institutions were invited to participate, and 17 institutions agreed to participate in the study. The treatment facilities have been randomized to offer one of the three following CRAFT formats:

1. CRAFT as individual format, consisting of six individual sessions with a therapist and a self-help book
2. CRAFT in an open group format. The groups start when two CSOs have contacted the treatment facility and will then continuously include new members, consisting of six group sessions with one or two therapists, in addition to the participants also getting a self-help book.
3. Control condition, consisting of CRAFT delivered in a self-directed format and using a self-help book only.

### Power calculation

Based on the data from Manuel et al. [20], we expect 40% of CSOs receiving self-help material and 60% of the CSOs receiving either Group CRAFT or Individual CRAFT to be able to motivate the drinkers to enter treatment. Furthermore, the effect of CRAFT is one-sided as the intervention of CRAFT cannot make the situation inferior for either the CSO or the drinker [8]. Based on these expectations, 106 participants in each group are needed to be able to detect a 20%-point difference with an α level at 5%, a power of 90% and an ICC of 0.05. As we foresee a dropout rate at approximately 10% and, furthermore, a loss to follow-ups rate at 10%, we need to include at least 135 CSOs in each group. This gives an expected total of 405 CSOs.

### Randomization and blinding

**Fig. 1:**
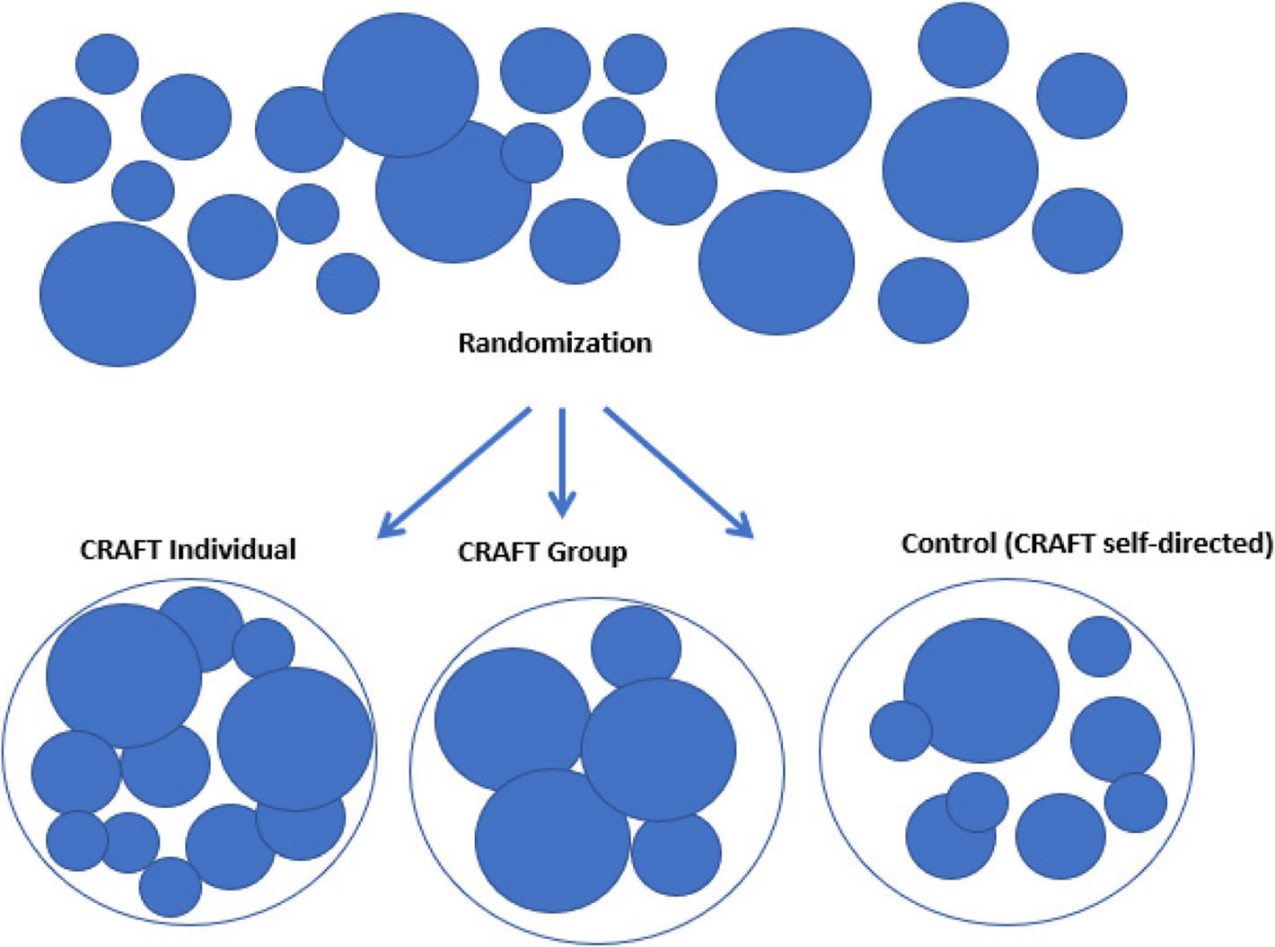
Cluster-randomization of treatment institutions

Consecutive CSOs, who contact a center randomized to conduct either individual CRAFT or open group CRAFT, will be offered the intervention within two weeks of an intake interview. The intervention consists of six sessions with 7–10 days between each session (Fig. 1).

Consecutive CSOs, who contact a center randomized to the control condition (self-directed CRAFT), will be offered self-help material, only. After three months, and when the primary outcome has been measured, the CSOs in this intervention format will, however, have the possibility of an individual follow-up session with a therapist to make sure that the CSOs are helping to move on.

As can be seen from the design, the control group consists of CSOs randomized to self-help intervention. The self-help intervention is chosen as control condition instead of either Treatment as usual or waiting list. Treatment as usual was disregarded since the interventions, currently being offered to the CSOs in the participating treatment centers, differ a lot one from the other, ranging from nothing, to brief advice on the phone, psycho-education, or personal support. A waiting list is disregarded as control condition since participants, waiting for an intervention or treatment, do not act ‘naturally’, but simply wait and become worse than they would have outside the study [30], potentially leading to an overestimation of the effectiveness of the experimental condition. Instead, offering self-help material to the control group serves as a minimal intervention and, for some CSOs, it may even be sufficiently helpful. Should that be the case, it would constitute an interesting finding and be added to the overall implementation of CRAFT nationwide.

### Participants

The participants of the study are concerned significant others (CSO) to a person with an AUD. There is no consensus definition on a CSO in the literature, but it could, for example, be a spouse, a daughter, a son, a cousin, a friend or a colleague. The CSO must fulfill the criteria listed below to be included in the study. The therapist, who includes the CSO, estimates whether the CSO fulfills the inclusion and exclusion criteria.

Inclusion criteria (CSO):

1. 18 years and older
2. Being a CSO to a person with AUD, who is not currently under treatment for an alcohol problem
3. Having the intention to maintain contact during the next 90 days
4. Having had regular contact with the PWAUD for the last 90 days (face-to-face contact for several hours on, at least, a weekly basis) or having the wish to re-establish a regular contact to PWAUD
5. Being prepared, at least to some extent, to support the problem drinker if he/she chooses to seek treatment

Exclusion criteria (CSO):

6. Suffering from dementia or other cognitive disorders
7. Not speaking Danish
8. Being psychotic or otherwise severely mentally ill
9. Have been in treatment for AUD in the last three months
10. Being concerned about a person who, according to the CSO, mainly uses illegal substances

### Recruitment

Informative leaflets and posters have been distributed to participating local authorities, who are committed to distribute the leaflets via social services departments, departments for children and adolescents, and to general practitioners. Additionally, we recruit participants through advertisements in local newspapers and via videos and posts on Facebook, linking to the participating alcohol treatment centers’ websites and their Facebook pages. Further, we inform about the project on national websites for counseling about alcohol problems such as www.hope.dk and the national telephone hotline ‘Alkolinjen’.

### Timeline

#### Interventions

##### Individual and group sessions

**Fig. 2:**
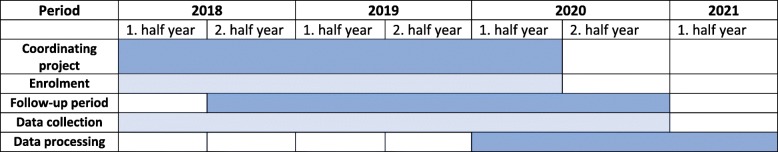
Project time-line

In this study the sessions are compressed to 6 sessions of one hour for individual CRAFT and two hours for group CRAFT in order to test if a shorter period is just as effective as 12–14 sessions (Fig. 2).

The group CRAFT and the individual CRAFT interventions cover the following topics [8]:

1. Training in recognition of early signs of domestic violence, particularly as new behavioural change techniques are introduced, intentionally designed to be experienced as negative by the PWAUD; development of a safety plan.
2. Development of a functional analysis to outline the triggers of the drinking problem, as well as the positive and negative consequences of it; training in identifying the CSO’s own unintentional role in the maintenance of the PWAUD’s using cycle.
3. Training in effective communication with the PWAUD. Training in appropriate and consistent use of positive reinforcement of the PWAUD’s non-using prosocial behaviour.
4. Training in positive reinforcement. Learning to reinforce clean and sober behaviour by using small rewards.
5. Training the withdrawal of reinforcement at times of drinking episodes in order to allow for the natural negative consequences of PWAUDs’ using behaviour.
6. Help to identify the CSO’s own areas of life dissatisfaction and training the development of specific plans for addressing that dissatisfaction and in rewarding themselves more often.
7. Training the methods on how and when to suggest treatment to a PWAUD. Development of a “rapid intake” plan. Working with how to handle disappointments in a fruitful way.

##### Self-directed CRAFT format (control)

CSOs, randomized to the control condition, will receive the self-help material, only, and are considered controls for the first three months after enrollment. The self-help material is a Danish book [31], inspired by the American CRAFT self-help book “Get Your Loved One Sober” [32] and the German self-help book “Strategien zur Selbsthilfe für Angehörige von Menschen mit Alkoholproblemen, Der Community Reinforcement Ansatz: das Familien-Training (CRAFT)” [33]. In addition, we added a chapter about what alcohol dependence is like, in addition to describing alcohol treatment, how it is organized and how it can be sought. The CRAFT studies, so far, indicate that the effect of the face-to-face intervention is mainly seen either during the intervention or shortly after completion of the intervention. In other words: empowering the CSOs seems to lead to rather immediate changes in relation to treatment-seeking behaviour among PWAUD.

Since we expect a higher impact from the face-to-face intervention than from the control condition, all participants in the control condition have the option for a face-to-face session with therapist three months after enrollment in the study, in order to ensure that the participants are feeling sufficiently helped. The additional face-to face session for the control group is for ethical reasons and postponed in time, in order not to disturb the testing of the interventions.

#### Participating therapist and training

The therapists delivering the CRAFT intervention will be staff from the participating treatment centers. Typically, the therapists are educated nurses or social workers with extensive experience in the treatment of alcohol abuse disorders. Therapists from the participating centers, randomized to deliver either group-based or individual CRAFT, have received three days free training in CRAFT, funded by the present project. To avoid disappointment if a participating center is randomized to deliver self-directed CRAFT, the control intervention, and in order to add to the overall implementation of CRAFT nationwide, therapists will be offered identical training free of charge when the enrollment of participants is completed.

During the study, all therapists, delivering the experimental intervention, will receive feedback on recordings of their intervention performance. All face-to-face intervention sessions will be recorded, and feedback will be given on randomly picked recordings.

#### Instruments and data collection

Data will be collected at baseline (t0), three months (t1), and six months (t2) by means of an iPad (baseline) and by a Web-based battery of questionnaires or by telephone interview (at follow-ups). The participants receive up to three reminders for the follow-up questionnaire until they have answered. Data on whether and when PWAUD start treatment, will be collected from the CSO after three and six months after completion of enrollment of CSOs.

The questionnaire will consist of the following instruments (Table 1):

**Table 1:** Instruments at baseline, first follow-up and second follow-up

	Baseline	First follow-up after 3 months from baseline (t_1_)	Second follow-up after 6 months from baseline (t_2_)
Instruments			
Demographic information	X		
Information on whether the drinker has sought treatment (primary outcome)		X	X
Audit about use of Alcohol [34]	X	X	X
Quality of life [35]	X	X	X
Self-reported number of days with sick leave within the last 30 days	X	X	X
Time spent with the problems drinker [36]	X	X	X
The drinker’s use of alcohol (according to the CSO) [36]	X	X	X
PHQ9_Danish [37]	X	X	X
Coping Questionnaire 30 items form [38]	X	X	X
Family Member Impact Questionnaire [38]	X	X	X
Personal Happiness Scale [39]	X	X	X
Satisfaction with the intervention received [40]		X	X

### Outcome measures

#### Primary outcome

The primary outcome is the proportion of drinkers, who enter alcohol treatment from baseline and until three months after their CSOs’ enrollment in the CRAFT study.

#### Secondary outcomes

1. Changes in the anxiety and depression symptoms of CSOs following CRAFT intervention
2. Improvement in the relationship between the CSO and PWAUD before and after CRAFT intervention
3. Self-reported changes in number of sick leave days for CSOs from 6 months before to 6 months after enrollment in the study
4. Changes in quality of life among CSOs

### Statistical analyses

Data will be analyzed using a mixed effect logistic regression model, mixed effects Poisson regression, and univariate statistical models, including t-tests, and chi2-tests, will be used for descriptive statistics. For some of the quantitative secondary outcomes a mixed effect model (for normally distributed data) or a random effect quantile regression model will be used. Strategy for missing data will be based on multiple imputations with special emphasis on the sensitivity of the results of various imputation schemes, as the missing mechanism is likely to be missing not at random.

### Ethics

In view of the fact that the CRAFT intervention has proven highly effective in the US, and since living close to a problem drinker is such a burden to the individual and, finally, because the Danish National Guidelines strongly recommend the implementation of CRAFT, we find it un-ethical and problematical not to offer CRAFT in some form to all the participants, even to the control condition. However, since we expect less effect of the control condition (CRAFT as self-help material), for ethical reasons, the participants in the control group will be offered an individual CRAFT session three month after enrollment. Training significant others in new communication strategies and new ways of acting, in relation to the problem drinker, may lead to increased tension in the family set-up. Domestic violence is already relatively more frequent in families with problem drinking, thus, an increased risk may be anticipated. It is, therefore, highly important to address the risk of domestic violence in therapy. Addressing this risk is mandatory in the interventions and includes developing safety plans together with the CSO. It is, however, important to bear in mind that the risk of abuse and suffering, on the part of the CSOs, is even higher if the problem drinking continues unaddressed. We expect no risks or side-effects for the participants.

### Data management

Data will be collected in Research Electronic Data Capture (REDCap). Baseline data will be collected directly on an iPad. Data from the follow-up points (three months and six months after baseline) will be collected by means of Web-based questionnaires. All data will be handled and stored by Odense Patient Data Explorative Network (OPEN) [43] and safe SharePoint. The researchers RH, RB, ASN, CE, one data manager, and one statistician will have access to the data and final dataset. The researcher RH and RB make interim analysis and present interim analysis for the scientific advisory board. The researcher RH and RB are responsible for recording adverse events and other unintended effects of the trial intervention.

### Dissemination policy

Only the investigators have the right to publish the results. Firstly, the results will be published in international peer-reviewed journals. Afterwards, the results will be shared in the press and presented at international conferences. Moreover, some of the results will be a part of a Ph.D. thesis. The results will also be communicated to the participants and the therapists in the study.

### Organization, administration and oversight

The study will be carried out by the Unit of Clinical Alcohol Research (UCAR), The Clinical Research Institute, and The University of Southern Denmark. UCAR is governed by two complementary functions: A Research Office and a Scientific Advisory board.

The UCAR Research Office manages the daily research operations of UCAR and comprises UCAR’s director, the leading Principal Investigators and administrative staff. The Research Office is responsible for the general administration of research projects, including the present study, and provides specialized services to researchers. Regular meetings will take place between The Research Office, key-persons and leaders of the treatment centres, to inform about progress of the study. Furthermore, a monthly newsletter will be sent to all parties involved in the study.

The Scientific Advisory board meets three times a year, to discuss the progress in study. The scientific Advisory board have the right to make the final decision to terminate the project. The sponsors have no influence on the process.

### Pilot phase

The first four months of the inclusion-period (from January 2018) have been considered as the pilot phase, in which all procedures, from inclusion of the first participants in the study to the first follow-up interview after 3 months, are tested and evaluated. Together with the participating treatment centers, we have evaluated all the procedures. We have also estimated the duration of the entire inclusion period of 405 participants. Regardless that we only have included 60 CSOs in the first four months, we decided to invite more treatment institutions to the project. This resulted in an additional seven institutions to be trained, randomized and commence the inclusion of CSOs from the first of September 2018.

## Discussion

The aim of this study is to investigate whether CSOs assigned to individual CRAFT or group CRAFT would be more able to motivate their drinking relative to enter treatment, compared to CSOs randomly assigned to the control condition (self-directed CRAFT) and, furthermore, to investigate whether six-weeks group CRAFT improves the quality of life and psychological functioning of CSOs significantly more than individual CRAFT and self-directed CRAFT.

We expect to find a higher improvement in the quality of life for the CSOs receiving group CRAFT than the ones receiving individual CRAFT, since the CSOs in the group CRAFT benefit from the dynamics that occur in a group of like-minded individuals. Being part of a group creates a sense of mutual recognition and may lower the feeling of isolation and shame among CSOs [42]. Individual sessions may, however, be easier to attend, offer more flexibility, and may allow the CSO to work more freely with what is perceived to be most important. We would like to study if there is a difference in life quality for the CSOs whose relatives enter treatment and those who do not (self-directed CRAFT). This study is also the first to study group CRAFT in Europe and the first study using open group format. CRAFT in a group format may be a relatively cheaper solution [20]. However, individual sessions may be easier to organize in smaller facilities, if waiting lists are to be avoided. If the self-help material proves to be just as effective as other types of intervention it would be a low-cost intervention and easy to implement nationwide.

Alcohol abuse is high taboo in Denmark and CSOs often feel ashamed of the abuse in the family. Therefore, we know it can be hard to attract the CSOs. The knowledge about the free public alcohol treatment in Denmark is rather low. Compared to the USA, where drinking persons were offered alcohol treatment free of charge if the CSO participated in the CRAFT studies, the incentive was higher for both CSO and for PWAUD. Since treatment for alcohol use disorder in Denmark is tax funded, free to seek and easily available, participation in this CRAFT study cannot be used as a special opportunity for free treatment and, therefore, we may not expect to replicate the high engagement rates from the US studies [26]. We might also be aware of the fact that not all CSO find it realistic that the drinking person will ever enter treatment. It can, however, rather be an aim for the CSO to increase their own quality of life and their relationship with the drinking person, only.

We expect this study to provide evidence on the efficacy of CRAFT in Denmark and provide an answer as to whether one of the three CRAFT methods are more effective than the other.

## Abbreviations

ANF: Al-Anon/Nar-anon
AUD: Alcohol Use Disorder
CRAFT: Community Reinforcement and Family Training
CSO: Concerned significant other
GP: General practitioner
OPEN: Odense Patient Data Explorative Network
PWAUD: People with Alcohol Use Disorder
REDCap: Research Electronic Data Capture
TEnt: Treatment Entry Training
UCAR: Unit of Clinical Alcohol Research
WHO: World Health Organization
